# Oxygen Vacancy Formation and Migration within the Antiphase Boundaries in Lanthanum Scandate-Based Oxides: Computational Study

**DOI:** 10.3390/ma15072695

**Published:** 2022-04-06

**Authors:** Yuri A. Mastrikov, Denis Gryaznov, Maksim N. Sokolov, Guntars Zvejnieks, Anatoli I. Popov, Roberts I. Eglitis, Eugene A. Kotomin, Maxim V. Ananyev

**Affiliations:** 1 Institute of Solid State physics, University of Latvia, Kengaraga 8, LV-1063 Riga, Latvia; gryaznov@cfi.lu.lv (D.G.); maksims.sokolovs@cfi.lu.lv (M.N.S.); guntars.zvejnieks@cfi.lu.lv (G.Z.); anatolijs.popovs@cfi.lu.lv (A.I.P.); roberts.eglitis@cfi.lu.lv (R.I.E.); jevgenijs.kotomins@cfi.lu.lv (E.A.K.); 2 Max Planck Institute for Solid State Research, Heisenbergstraße. 1, D-70569 Stuttgart, Germany; 3 Federal State Research and Design Institute of Rare Metal Industry (Giredmet JSC), Electrodnaya Street 2, 111524 Moscow, Russia; m.ananyev@mail.ru

**Keywords:** La_1−*x*_Sr*_x_*ScO_3−*δ*_, lanthanum scandate, perovskite, antiphase boundaries, oxygen vacancy, oxygen transport, DFT

## Abstract

The atomic structure of antiphase boundaries in Sr-doped lanthanum scandate (La_1−*x*_Sr*_x_*ScO_3−*δ*_) perovskite, promising as the proton conductor, was modelled by means of DFT method. Two structural types of interfaces formed by structural octahedral coupling were constructed: edge- and face-shared. The energetic stability of these two interfaces was investigated. The mechanisms of oxygen vacancy formation and migration in both types of interfaces were modelled. It was shown that both interfaces are structurally stable and facilitate oxygen ionic migration. Oxygen vacancy formation energy in interfaces is lower than that in the regular structure, which favours the oxygen vacancy segregation within such interfaces. The calculated energy profile suggests that both types of interfaces are advantageous for oxygen ion migration in the material.

## 1. Introduction

Sr-doped lanthanum scandate—La_1−*x*_Sr*_x_*ScO_3−*δ*_ (LSS)—is a well-known proton conductor used in protonic–ceramic fuel cells (PCFC) [1]. Crystalline structure, electrical conductivity, water incorporation, and oxygen and hydrogen diffusivities of lanthanum-strontium scandate were studied in [2,3,4,5].

Contrary to acceptor-doped Ba(Zr,Ce)O_3_ materials [6,7], La_1−*x*_Sr*_x_*ScO_3−*δ*_ oxides demonstrate high stability and protonic conductivity not only in water-containing but also in reduced and carbon-containing atmospheres of H_2_ and CH_4_ [8,9]. Recent experimental studies suggest the presence of various defects therein, including planar defects and antiphase boundaries (APB), which affect the material’s performance [10]. In that study, defects, represented as straight or arbitrarily curved lines that did not extend beyond the grain boundaries, were identified as π-type antiphase boundaries. The boundaries between antiphase domains are experimentally observed as local structural inhomogeneities within the LSS grains of the polycrystalline specimens. The simplest model for the structure of such boundaries can include different types of structural octahedral junction: not by a vertex, as in a regular perovskite structure, but by edges and by faces. Such junctions give rise to new interfaces with differences from the regular oxygen positions within them. The defect-induced structures may strongly influence oxygen ionic transport, governed by the oxygen vacancies formation and migration energies.

The stacking disorder can be considered as a dislocation, as an APB, or even as a different phase. For example, edge-shared structures in SrTiO_3_ (STO) are seen as dislocations [11]. Face-sharing of oxygen octahedra give rise to “twinned” structures in Ba(Co, Nb)_1−*δ*_O_3_ [12] or various hexagonal perovskites [13]. Proton uptake may occur via the dissociative adsorption of H_2_O into the oxygen vacancies, forming protonic defects (acid-based hydration reaction):

(1) $$H_{2}O^{\left(g\right)}+V_{O}^{••}+O_{O}^{\times }\to 2{\mathrm{OH}}_{O}^{•}$$

where $V_{O}^{••}$ is oxygen vacancy, $O_{O}^{\times }$ oxygen ion at the regular site, and $2{\mathrm{OH}}_{O}^{•}$ is the hydroxide ion occupying the oxygen site in Kröger–Vink notation [14].

As follows Equation (1), the reaction requires a constant supply of oxygen vacancies from the bulk to the surface of the material. The critical parameters for this process are oxygen vacancy formation $E_{f}$ and $E_{m}$ migration energies, calculated in this study as defined in Equations (2) and (3):

(2) $$E_{f}=E_{vac}+\frac{1}{2}E_{O_{2}}-E_{bulk}$$

(3) $$E_{m}=max\left(E_{TS}-E_{vac_{i}}\right)$$

where $E_{TS}$ is the energy of the system in the transition state $E_{vac_{i}}$ energy of the system in the initial and in the final states before (after) the defect jump.

In the present computational study, we designed two types of antiphase boundaries in La_1−*x*_S*_x_*rScO_3−*δ*_, namely edge- and face-shared, and simulated the oxygen migration process therein in order to estimate the role of the oxygen vacancies formation and migration near the APB in the total ionic transport in lanthanum scandate-based oxides.

## 2. Model

In La_1−*x*_Sr*_x_*ScO_3−*δ*_, oxygen stoichiometry *δ* is controlled by Sr concentration *x*. Taking into account the formal charges, only the most optimal value of δ is *x*/2. In this case, two electrons of oxygen vacancy recombinate with two electron holes created by two Sr cations. Lanthanum scandate LaScO_3_ is a perovskite-type material with a strongly pronounced orthorhombic distortion (Space group *Pnma*, No. 62) [15]. There is one non-equivalent position for La and Sc (4*c* and 4*a*, respectively) and two non-equivalent positions for O—apical and equatorial (4*c* and 8*d*, respectively). Sr-doped material has the same structure [4,16,17]. All structures under study were completely optimized by reducing the external pressure to < 1 kbar and forces on atoms to 0.03 eV/Å.

${\mathrm{Sr}}_{\mathrm{La}}^{\prime }$ substitution gives rise to either electron holes on oxygen *p*-orbitals or oxygen vacancies ($V_{o}^{••}$). Doping by Sr as well as oxygen vacancy formation alone changes the electroneutrality of the system, which has to be compensated. Such a compensation of ${\mathrm{Sr}}_{\mathrm{La}}^{\prime }$ by $V_{o}^{••}$ can be presented as following:

(4) $$2\mathrm{SrO}\to 2{\mathrm{Sr}}_{\mathrm{La}}^{\prime }+V_{o}^{••}+2O_{O}^{\times }$$

A vacancy compensation mechanism is likely to prevail at high temperatures. In our neutral supercell model, the substitution Sr_La_ introduces an electron hole on oxygen *p*-orbitals. Oxygen vacancy formation in *AB*O_3_ perovkite-type materials causes either localization of two electrons of the missing O^2−^ ion at the vacancy site (F-centre), their localization on 3*d*-orbitals of the nearest transition metal *B*-cations, or their distribution on *p*-orbitals of oxygen anions. In case of lanthanum scandate, without Sr doping, localization of extra electrons on Sc cations is energetically very unfavourable. Thus, the formation of F-centres is quite anticipated. In the presence of Sr, F-centre, accepting two electron holes from oxygen *p*-orbitals, transforms into F^++^-centre. Therefore, for *x*/*δ* = 2 in La_1−*x*_Sr*_x_*ScO_3−*δ*_, all electron holes are compensated by electrons.

For the systems with oxygen vacancy, only atomic coordinates were optimized, with the lattice constants kept fixed as in the defect-free optimized structure.

## 3. Method

For modelling, we deployed the first principles DFT method as implemented in the computer code VASP [18] with GGA-PBE [19] exchange-correlation functional. The plane-wave basis kinetic energy cut-off was set at 400 eV, which corresponds the largest cut-off energy of the potentials used. The details of core PAW [20] potentials are given in Table 1. The cell extensions in units of lattice vectors and *k*-point sampling [21] in the Brillouin zone are given in Table 2. Extensions of transformed cells are given in basis vectors of the original (4 *AB*O_3_ units) cell in *Pnma* setting. *k*-Point mesh was generated relative to the transformed cell.

### Antiphase Boundaries

**Figure 1:**
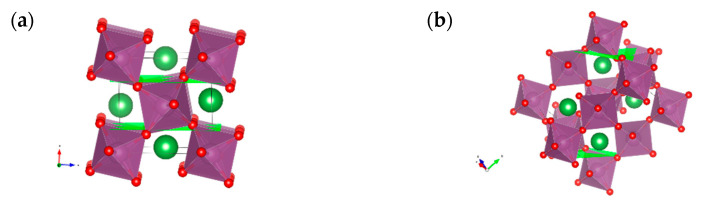

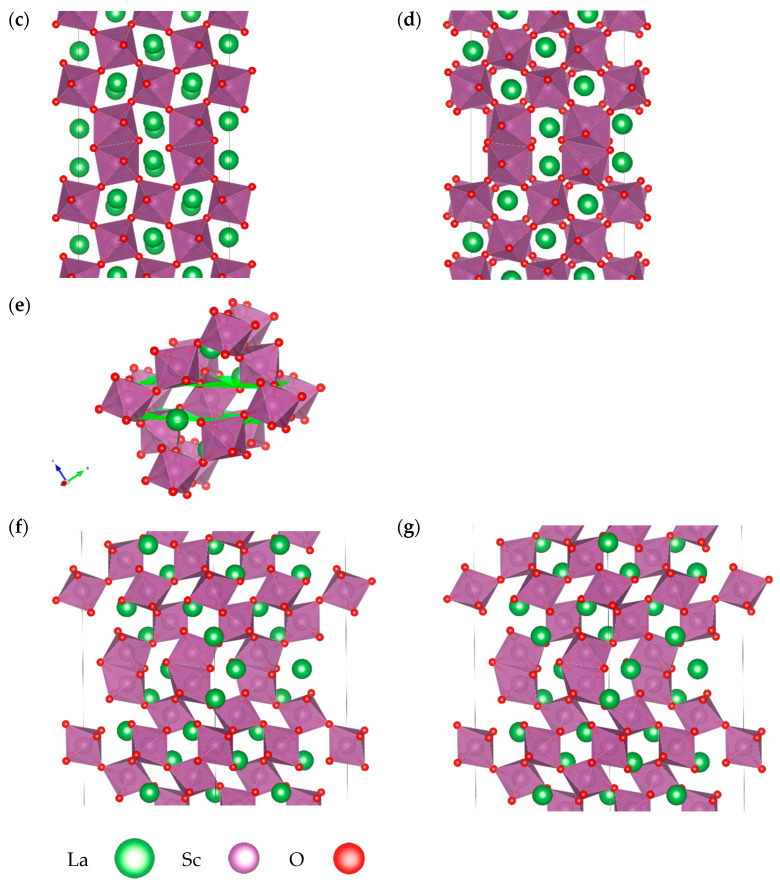
(100) (**a**), (121) (**b**), and (011) (**e**) planes and, respectively, antiphase boundaries in them (**c**,**d**,**f**,**g**).

APBs were designed as a stacking irregularity [22]. The interfaces with edge-shared oxygen octahedra were made by removal one or more crystallographic planes of (100) and (121) orientation, followed by closing the gap and merging the corresponding surfaces (Figure 1a–d). In the (100) interface, the merge is completed by equatorial oxygen atoms (Figure 1a,c). An alternative orientation for equatorial oxygen atoms edge sharing, (001) fails to provide a reasonable structural match. In (121) interface, both types of oxygen atoms are present—apical and equatorial (Figure 1b,d).

Making face-shared oxygen octahedra interfaces, besides removal of the (011) crystallographic planes (Figure 1e), requires reorientation and shift of one phase relative to another (Table 3). Due to the difference in mutual orientations of the phases, two non-equivalent interfaces of the same (011) orientation were proposed (Figure 1f,g).

In order to make interface formation energies for different interfaces directly comparable, and since lattice parameters are method dependent, the values are given relative to quasi-cubic surface unit *a*_0_^2^.

The structures were visualized by the VESTA [23] software. The same software was used for X-ray diffraction (XRD) simulations with the Cu-*K*α source with the wave lengths f_E_ 1.54059 and 1.54432 Å and intensities of 1 and 0.5, respectively.

## 4. Results

The interface formation energies are listed in Table 4. Small formation energies suggest that both types of APB, edge- and face-shared, can exist in the material. The merge along the equatorial line (100) is clearly energetically more favourable than that along the meridional one (121). Due to the dependence of the lattice constants on a particular computational method, energies are normalized to the perovskite structural units. That makes possible a direct comparison with the results of interface/surface calculations of LSS as well as other perovskite-type materials obtained by different computational methods.

**Figure 2:**
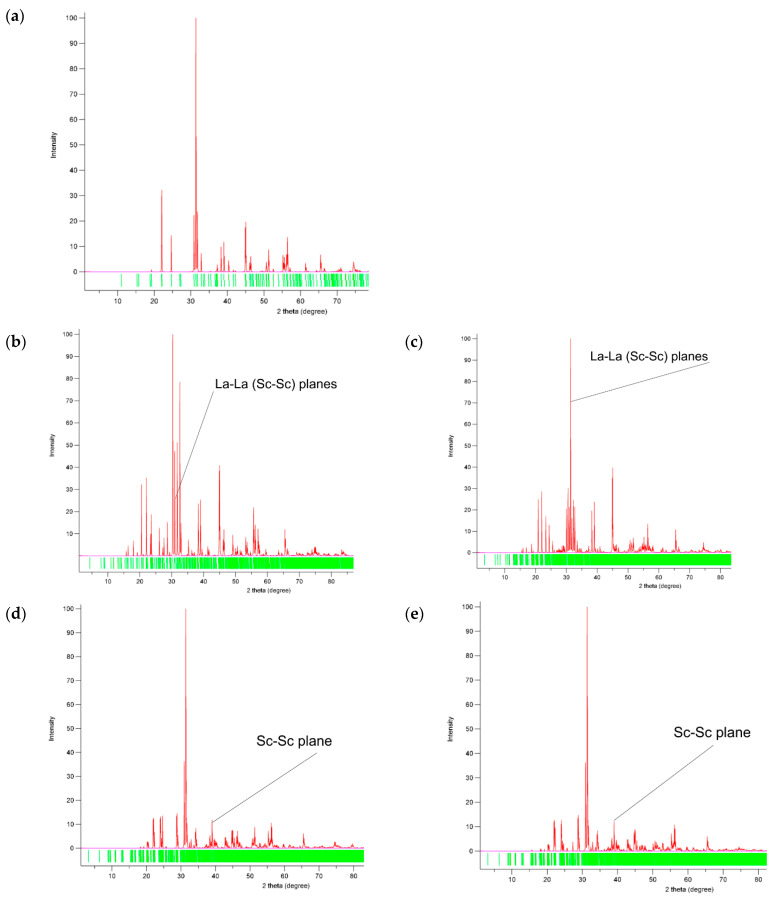
Simulated powder diffraction (XRD) pattern with the source of f_E_ 1.54059 and 1.54432 Å, with intensities of 1 and 0.5, respectively, for (**a**) ideal bulk, (**b**) (100) edge-shared interface, (**c**) (121) edge-shared interface, (**d**) (011) face-shared interface (Figure 1f), and (**e**) (011) face-shared interface (Figure 1g).

Each interface produces its own powder XRD pattern (Figure 2), clearly distinct from that of the ideal bulk (Figure 2a). The difference is clearly seen even between structurally very similar two face-shared (011) interfaces (Figure 2d,e). The presented XRD patterns indicate that the existence of the antiphase boundaries can experimentally manifest itself on the local diffraction pattern as a structural inhomogeneity with a lower symmetry. The view of the patterns show that locally, these inhomogeneities caused by the APBs can be mixed up with a superstructure domains of higher-order lanthanum–strontium scandates: (SrO)*_n_*_−1_(LaScO_3_)*_n_* [24].

As seen in Figure 2, the characteristic peaks for edge-shared interfaces can hardly be discriminated from the major peaks of the regular structure, whereas face-shared structure produces a well-pronounced peak at about 2Θ = 39°, which corresponds to the interface-unique smallest distance between the Sc-Sc planes (Figure 1f,g).

Oxygen vacancy modelling was performed by introducing a single vacancy into the system, and electron charge was compensated by two Sr_La_ cations—La_1−*x*_Sr*_x_*ScO_3−*x*/2_. For edge-shared interface, the composition of the materials is La_0.93_Sr_0.07_ScO_3−0.04_ (*x* = 1/14) and for face shared—La_0.96_Sr_0.04_ScO_3−0.02_ (*x* = 1/48).

**Figure 3:**
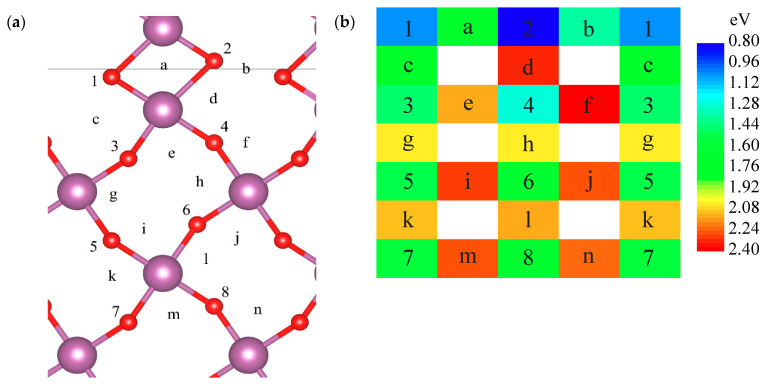
Oxygen vacancy formation energy (numbers) and transition states (letters) within the (100) edge-shared (Figure 1c) interface (solid horizontal line) and in the bulk. (**a**) Indexed crystal structure, (010) plane, and (**b**) schematic view with coloured energy states, relative to the ideal interface and 1/2O_2_ (Equation (2)).

Oxygen migration process in edge-shared interface was modelled for the (100) configuration only (Figure 1c and Figure 3). Vacancy was placed in non-equivalent equatorial oxygen positions within the same crystallographic (010) plane. Only equatorial–equatorial jumps were considered. Vacancy formation energies at the interface are at least by 0.4 eV smaller than the reference values (1.6 eV) (Table 5). The APB irregularity of the structure gives rise to two distinct activation barriers—labelled *a* and *b* (Table 5). Barrier *a* corresponds to the jump along the shared edge (1.0 eV). At the same time, adjacent to it, barrier *b* is the lowest one (0.5 eV) and is characterized by a larger distance to the nearest Sc atoms than any other O—O-bond. Similar results are expected for the (121) edge-shared interface (Figure 1d).

**Figure 4:**
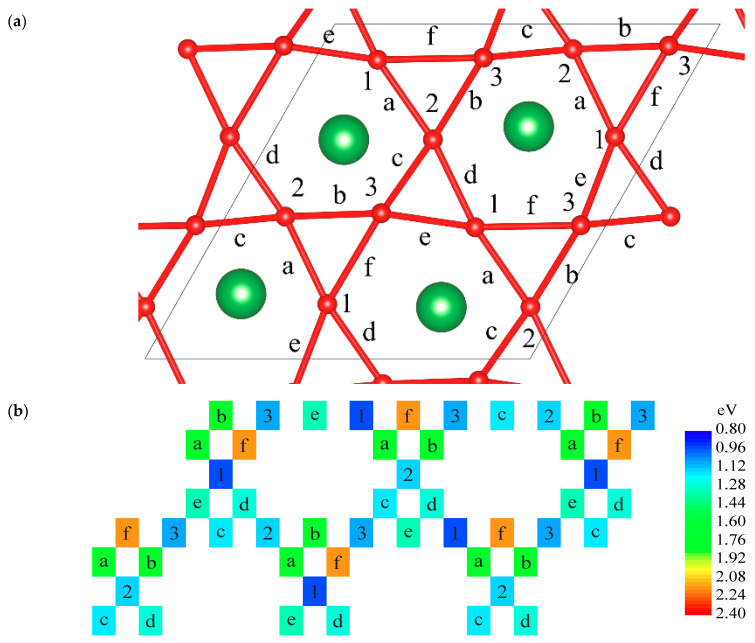
Oxygen vacancy formation energy (numbers) and transition states (letters) within the (011) interface, relative to the ideal interface and 1/2O_2_ (Equation (2)) (Figure 1d). (**a**) Indexed crystal structure and (**b**) schematic view with coloured energy states, relative to the ideal interface and 1/2O_2_.

For (011) APB, oxygen migration was modelled within the plane of the interface (Figure 4). Just like for the (100) interface, vacancy formation energies for (011) APB are lower by 0.4 eV (Table 6). For the reference, two vacancies were modelled at the largest distance in the system from the (011) APB. All the formations, TS, as well as activation energies are higher than those within the interface.

Performed DFT calculations showed that the complex interfaces forming extended net-like defect structures within a grain can lead to the emergence of new migration pathways for oxygen ions. Since proton defects in LSS are also associated with oxygen migration, the presence or absence of oxygen deficiency and the associated presence of such types of defects can directly affect the migration of protons. It was found experimentally that the apparent activation energy of hydrogen diffusion in the atmosphere of water and the atmosphere of molecular hydrogen differ by 0.2–0.3 eV [4]. One of the reasons for this difference may be the migration of oxygen ions and protons along the interfaces that exist in the atmosphere of molecular (dry) hydrogen since its incorporation into the scandate structure does not lead to the healing of oxygen vacancies [8] in contrast to the process of migration of protons incorporated from water atmospheres [3].

## 5. Conclusions

Two distinct types of possible antiphase boundaries in lanthanum scandate-based oxides doped with strontium were computationally designed and investigated—edge- and face-shared oxygen octahedra structures. For each interface type, the most energetically stable structures were found. Such interfaces may exist in all perovskite-type crystals and can be identified by local diffraction methods. The oxygen vacancy formation energies for the edge-shared interface (100) are 0.8–1.0 eV, within the computational method applied, which is lower than in the regular structure reference value: 1.6 eV. Two distinct oxygen vacancy jumps with high activation energy (1.0 eV), over the shared edge, and low activation energy (0.5 eV), between shared edges, were identified. For face-shared (011) interface, vacancy formation energies are much lower (≈1.0–1.1 eV) than those at the distance from the interface (5.6–5.7 eV).

The energy state mapping for both types of interfaces shows that the more favourable than in the regular structure states are located within the interface. This has to be true regardless of the computational method chosen; yet, the absolute values of the calculated vacancy formation and migration energies could be different. The performed calculations indicate that it is energetically favourable for oxygen vacancies to segregate to antiphase boundaries. Most likely, this is true for all isostructural materials despite the difference in absolute values of oxygen vacancy formation and migration energies.

Performed modelling gives a solid basis for experimental detection and investigation of antiphase boundaries in lanthanum scandates as well as in other perovkite-type materials.

## Figures and Tables

**Table 1:** PAW core potentials.

Potentials	Valence Electrons	E_cutoff_, eV
La	5*s*^2^5*p*^6^5*d*^1^6*s*^2^	219.292
Sr	4*s*^2^4*p*^6^5*s*^2^	229.353
Sc	3*d*^1^4*s*^2^	154.763
O	2*s*^2^2*p*^4^	400.000

**Table 2:** Interface cells parameters—extensions in units of the original lattice vectors (*Pnma* setting) and *k*-points, relative to the new basis.

Structure	Extensions	*AB*O_3_ Units	*k*-Points
Bulk	$\left(\begin{array}{ccc} 2 & 0 & 0\\ 1 & 1 & 1\\ 1 & 1 & -1 \end{array}\right)$	16	2 × 2 × 2
Interface (100)	$\left(\begin{array}{ccc} 3.5 & 0 & 0\\ 0 & 1 & 0\\ 0 & 0 & 2 \end{array}\right)$	128	4 × 8 × 6
Interface (121)	$\left(\begin{array}{ccc} 2.25 & 2.25 & 2.25\\ 1 & -1 & 1\\ 1 & 0 & -1 \end{array}\right)$	128	4 × 6 × 8
Interface (011)	$\left(\begin{array}{ccc} 1 & 1 & -1\\ -1 & 1 & -1\\ 0 & 2 & 4 \end{array}\right)$	96	4 × 4 × 2

**Table 3:** Transformation of one phase in (011) relative to another phase; (a) Figure 1f and (b) Figure 1g.

(a)	**Rotation**	**Shift**
	$\left(\begin{array}{ccc} 0 & 1 & 0\\ 1 & 0 & 0\\ 0 & 0 & -1 \end{array}\right)$	−1/6
		−1/6
		−1/4
(b)	**Rotation**	**Shift**
	$\left(\begin{array}{ccc} 1 & 0 & 0\\ 0 & 1 & 0\\ 0 & 0 & -1 \end{array}\right)$	−1/6
		−1/6
		−1/6

**Table 4:** Calculated relative to the ideal LaScO_3_ bulk, APB interface energy in eV per surface unit.

Parameter	Edge-	Edge-	Face- *	Face- **
	Shared Interface			
Interface orientation	(100)	(121)	(011) *	(011) **
Interface formation energy, eV (per quasi-cubic *a*_0_^2^ unit)	0.46	1.13	0.66	0.75
Interface unit	2O	2O	3O	3O
Interface unit area, in quasi-cubic *a*_0_^2^ units	$\sqrt{2}$	$\sqrt{2}$	$\sqrt{3}$	$\sqrt{3}$
APB concentration, %	14	11	17	17

* Figure 1f, Table 3a; ** Figure 1g, Table 3b.

**Table 5:** Oxygen vacancy formation (Equation (2)) and activation (Equation (3)) energy, as in Figure 3.

1	a	2	b	1.03	1.00	0.80	0.54
c		d		0.65		1.52	
3	e	4	f	1.44	0.88	1.27	1.12
g		h		0.58		0.77	
5	i	6	j	1.52	0.76	1.57	0.72
k		l		0.56		0.56	
7 *	m *	8 *	n *	1.55	0.69	1.57	0.68

*—Reference values for the system.

**Table 6:** Oxygen vacancy formation energy, as in Figure 4. Numbered stable states (Equation (2)) and activation (Equation (3)) energy, in eV.

***V*_o_**	**Formation Energy, eV**	**Transition State**	**Activation Energy, eV**
1	0.95	a	0.68
2	1.12	b	0.68
3	1.08	c	0.10
		d	1.27
		e	1.33
		f	1.20
**Reference values**	**Formation energy, eV**	**Transition State**	**Activation energy, eV**
Apical	5.56	Apical–Equatorial 6.33 eV	0.77
Equatorial	5.67		
